# Impact of artificial intelligence on radiology: a EuroAIM survey among members of the European Society of Radiology

**DOI:** 10.1186/s13244-019-0798-3

**Published:** 2019-10-31

**Authors:** European Society of Radiology (ESR), Marina Codari, Luca Melazzini, Sergey P. Morozov, Cornelis C. van Kuijk, Luca M. Sconfienza, Francesco Sardanelli

**Affiliations:** Vienna, Austria

**Keywords:** Artificial Intelligence, Machine Learning, Radiologists, Radiology, Surveys and Questionnaires

## Abstract

We report the results of a survey conducted among ESR members in November and December 2018, asking for expectations about artificial intelligence (AI) in 5–10 years. Of 24,000 ESR members contacted, 675 (2.8%) completed the survey, 454 males (67%), 555 (82%) working at academic/public hospitals. AI impact was mostly expected (≥ 30% of responders) on breast, oncologic, thoracic, and neuro imaging, mainly involving mammography, computed tomography, and magnetic resonance. Responders foresee AI impact on: job opportunities (375/675, 56%), 218/375 (58%) expecting increase, 157/375 (42%) reduction; reporting workload (504/675, 75%), 256/504 (51%) expecting reduction, 248/504 (49%) increase; radiologist’s profile, becoming more clinical (364/675, 54%) and more subspecialised (283/675, 42%). For 374/675 responders (55%) AI-only reports would be not accepted by patients, for 79/675 (12%) accepted, for 222/675 (33%) it is too early to answer. For 275/675 responders (41%) AI will make the radiologist-patient relation more interactive, for 140/675 (21%) more impersonal, for 259/675 (38%) unchanged. If AI allows time saving, radiologists should interact more with clinicians (437/675, 65%) and/or patients (322/675, 48%). For all responders, involvement in AI-projects is welcome, with different roles: supervision (434/675, 64%), task definition (359/675, 53%), image labelling (197/675, 29%). Of 675 responders, 321 (48%) do not currently use AI, 138 (20%) use AI, 205 (30%) are planning to do it. According to 277/675 responders (41%), radiologists will take responsibility for AI outcome, while 277/675 (41%) suggest shared responsibility with other professionals. To summarise, responders showed a general favourable attitude towards AI.

## Key points

- AI is mostly expected to impact breast, oncologic, thoracic, and neuro imaging.
- Mammography, computed tomography, and magnetic resonance are thought to be the most impacted imaging modalities.
- Expectations for AI impact on job opportunities and workload of radiologists include both increase and decrease.
- For more than half of responders, AI-only reports would be not accepted by patients.
- The working time potentially saved by AI should be used for a stronger interaction with clinicians and patients.

## Patient Summary

Radiology generates a huge amount of digital data as obtained images are included into patients’ clinical history for diagnosis, treatment planning, screening, follow up, or prognosis. Besides, the increasing use of computers and data has led to the successful utilisation of artificial intelligence (AI) to carry out several tasks for more accurate and up-to-date results. The European Society of Radiology (ESR) put together a survey aimed at determining the radiologists’ position towards these new technological innovations which could strongly impact their specialty.

About 2.8% of the 24,000 contacted ESR members answered the survey entirely. According to respondents, breast, oncologic, thoracic, and neuro imaging are the most likely to be strongly impacted by AI and new technological innovations, along with forms of imaging such as mammography, computed tomography (CT), and magnetic resonance imaging (MRI). More than half of respondents anticipate patients not to accept AI-only based reports while 12% expect patients to accept these reports and 33% stated it’s too early to answer this question. Meanwhile, it is still unclear if the responsibility of the AI systems outcomes will be borne by the radiologist alone or if it will fall under a shared responsibility scenario.

Radiologists’ job opportunities and workloads are expected to increase or decrease. The respondents believe that, should AI allow to save working time, the saved time should be used to provide for stronger interactions and increased communication with other clinicians (64.7% of respondents) and patients (47.7% of respondents). Of all radiologists who answered the survey, 41% believe the relationship between them and the patient will become more interactive while 21% claim it will become more impersonal. The remaining respondents believing the relationship will remain unchanged.

ESR respondents unanimously agreed that radiologists must play a leading role in developing and validating AI applications to medical imaging. It will require significant involvement of radiologists and use of their expertise to ensure the quality of data and effective transformation of development solution research into clinical practice. This, with the aim of improving the outcome for patients and the trust of patients in new developments. Moreover, accountability and ethical issues surrounding AI-systems would constitute a significant challenge that would require regulations at EU and international level. The survey also highlights the radiologists’ will to be educated on advantages and limitations, the clinical use, and technical methods of AI applications.

## Background

Among medical subspecialties, radiology is one of the most prolific generators of digital data. Each radiology department routinely generates a large and heterogeneous amount of data. Images daily acquired with different modalities, such as radiography, angiography, ultrasound (US), CT, MRI, or nuclear imaging, are integrated to patients’ clinical history to extract information with the aim of screening, diagnosis, treatment planning, and prognosis [1].

In this digitised environment, AI found its fertile ground for flourishing. Indeed, the recent achievements obtained thanks to the application of machine learning for medical image analysis have shaken the radiological world [2, 3]. Machine learning proved its applicability to different imaging modalities and radiological subspecialties. In particular, deep learning has emerged as a promising technique for processing medical imaging data, being employed for several tasks like image classification, segmentation, registration, and abnormality detection [4].

This data-driven revolution has the concrete possibility to drastically impact on radiology. The debate about how abrupt this impact will be is still open and controversial. Indeed, several opinion articles have been published reporting experts’ forecasts about the possible scenarios that radiologists will face in the next future. On the one hand, there are the optimists, who see in the AI an opportunity to enhance radiologist’s role in the healthcare system; on the other hand, there are the pessimists, who predict a relatively fast replacement of radiologists by AI systems [2, 5, 6].

Even though some of these scenarios may be considered as extreme, they all have a common characteristic: the involvement of radiologists as one of the designated users of AI-based systems. The controversial part is related to their active or passive role in this healthcare revolution. It is not easy to predict reactions and attitudes of radiologists to this technological innovation but we can investigate their current feeling about what the future will hold.

In this article, we report the results of an online survey aimed at investigating the feelings of members of the ESR about AI impact on their practice.

## Methods

We conducted an online survey entitled “Your expectations about AI in radiology”. The survey was composed of two subparts. The first part consisted of 7 questions related to respondent age, sex, radiology subspecialty, most frequently practiced techniques and working status, type of institution, and country (Table 1). No personal identifying data were collected.

**Table 1:** Multiple-choice questions related to respondent’s age, sex, radiology subspecialty, most frequently practiced techniques and working status, type of institution, and country

Question number	Topic	Answers	
		Maximum number	List
I	Status	1	Medical student, Resident, Radiologist, Engineer/Computer scientist, Physicist, Other
II	Working place	1	University/Teaching hospital, Hospital, Private practice, Private research centre, Private company, Other
III	Gender	1	Male, Female
IV	Age range	1	18–29 years; 30–39 years; 40–49 years; 50–59 years; 60–69 years; ≥ 70 years
V	Home country	1	Albania; Austria; Armenia; Belarus; Belgium; Bosnia and Herzegovina; Croatia; Cyprus; Czech Republic; Denmark; Estonia; Finland; France; Georgia; Germany; Greece; Hungary; Iceland; Ireland; Israel; Italy; Kazakhstan; Kosovo; Kyrgyzstan; Latvia; Lithuania; Luxembourg; Macedonia; Malta; Montenegro; Netherlands; Norway; Poland; Portugal; Romania; Russia; Serbia; Slovakia; Slovenia; Spain; Sweden; Switzerland; Turkey; Ukraine; United Kingdom; Uzbekistan
VI	Subspecialty	5	Breast; Cardiovascular; Emergency; Gastrointestinal/Abdominal; General; Head & Neck; Interventional; Molecular imaging/Nuclear; Musculoskeletal; Neuroradiology; Oncologic imaging; Paediatric; Thoracic; Urogenital
VII	Practiced techniques	5	Radiography; Mammography; Ultrasound; Angiography/Fluoroscopy; CT; MRI; PET/Nuclear; Hybrid imaging; DXA; Experimental imaging (animal models); Optical imaging; Other

The second part consisted of 15 multiple choice questions about user feelings/forecasts in respect to the advent of AI applications in radiological practice in the next 5–10 years. The detailed list of questions and possible answers is reported in Table 2. The survey was designed by the European Network for the Assessment of Imaging in Medicine (EuroAIM), a joint initiative of the European Institute for Biomedical Imaging Research (EIBIR), under the umbrella of the ESR Board of Directors.

**Table 2:** Multiple-choice questions about user feelings/forecasts in respect to the advent of artificial intelligence (AI) applications in radiological practice in the next 5–10 years

Question number	Topic	Answers	
		Maximum number	List
1	Which radiological subspecialties do you foresee will be more influenced by AI in the next 5–10 years?	3	Breast; Cardiovascular; Emergency; Gastrointestinal/Abdominal; General; Head & Neck; Interventional; Molecular imaging/Nuclear; Musculoskeletal; Neuroradiology; Oncologic imaging; Paediatric; Thoracic; Urogenital
2	Which techniques do you foresee will be the most important fields of AI-applications in the next 5–10 years?	3	Radiography, Mammography, Ultrasound, Angiography/Fluoroscopy, CT, MRI, PET/Nuclear, Hybrid imaging; DXA; Experimental imaging (animal models); Optical imaging; Other
3	Which of the following AI applications you think are more relevant as aids to radiological profession? (Up to 3 answers)	3	Imaging protocol optimisation; Image post-processing; Detection in asymptomatic subjects (screening); Detection of incidental findings; Lesion characterisation/diagnosis in symptomatic subjects; Staging/restaging in oncology; Support to structured reporting; Quantitative measure of imaging biomarkers; Prognosis; Other
4	Do you foresee an AI impact on professional radiologist’s life in terms of amount of job positions in the next 5–10 years?	1	No; Yes, job positions will be reduced; Yes, job positions will increase
5	In the next 5–10 years, the use of AI-based applications will make radiologists’ duties	1	More technical; More clinical; Unchanged; Other
6	Do you think that, in the next 5–10 years, the use of AI-based applications will help to report also examinations outside the field of subspecialisation?	1	No, radiologists will be more focused on radiology subspecialties; Yes, radiologists will be less focused on radiology subspecialties; The rate of dedication to subspecialties will remain unchanged
7	Do you foresee an AI impact on professional radiologist’s life in terms of total reporting workload in the next 5–10 years?	1	No; Yes, it will increase; Yes, it will be reduced
8	In the next 5–10 years, who will take the legal responsibility of AI-system output?	1	Radiologists; Other physicians (*e.g.*, clinicians asking for the imaging study); Developers of AI applications; Insurance companies; Shared responsibility; Other
9	In the next 5–10 years, will patients mostly accept a report from AI applications without supervision and approval by a physician?	1	Yes; No; Difficult to estimate at present
10	How will be the relationship between the radiologist and the patient because of AI introduction?	1	More impersonal; More interactive; Unchanged
11	What will be the role of radiologists in developing/validation AI applications to medical imaging?	2	None; Provide labelled images; Help in task definition; Develop AI-based applications; Supervise all stages needed to develop an AI-based application
12	Should radiologists be educated on	2	Technical methods, such as machine/deep learning algorithms; Advantages and limitations of AI applications; Clinical use of AI applications; How to get into the driver seat in using AI; How to avoid the use of AI applications; How to survive to AI revolution
13	If AI will allow to save working/reporting time, should radiologists use the saved time for interacting with	1	AI developers (*e.g.*, engineers, computer scientists); Other radiologists; Other clinicians; Patients; Administrators
14	Are you utilising AI-based products or services in your clinical practice?	1	Yes; No, but planning to utilise; No
15	Are you involved in research projects on AI-based application development?	1	Yes, testing; Yes, developing; No, but planning to be involved; No

The online survey was designed using SurveyMonkey platform (SurveyMonkey, San Mateo, CA, USA) and distributed by an email containing a non-serialised URL link. The email was sent to all ESR members. The survey was opened on November 6, 2018. The first email was then followed by two reminders on November 20 and 26, 2018. The survey was closed on December 8, 2018.

Results of ESR members responses to this survey were automatically recorded and processed using Excel (Microsoft, RedMond, WA, USA) and Matlab r2018b (MathWorks, Natick, MA, USA). Since all the questions deal with categorical variables, descriptive statistics was reported by means of frequencies and percentages. Moreover, correlation between ESR members and survey responders per country was estimated using the Spearman’s correlation coefficient.

## Results

### Demographics

The emails reached about 24,000 ESR members; 822 of them (3.4%) participated in the survey. A total of 675 of 822 responders completed the whole survey (82.1%). To make comparisons easier to interpret, we here report the results for 675 responders who represent 2.8% of the ESR members who received the emails. We found a significant positive correlation (ρ = 0.821, *p* < 0.001) between the number of survey responders per country and ESR members per country.

**Fig. 1:**
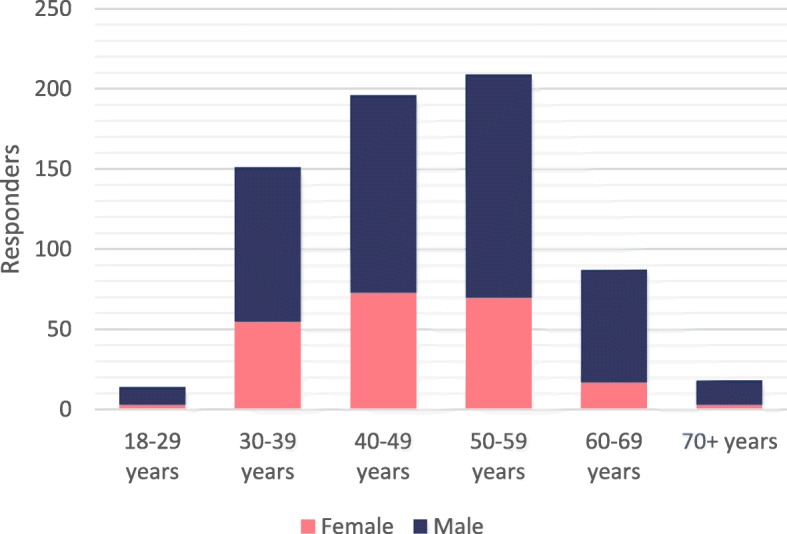
Distribution of survey responders according to age and sex

Detailed information about sex distribution according to age classes is reported in Fig. 1. Of the total 675 respondents, males were 454 (67.3%), females 221 (32.7%).

Among survey responders, 635 (94.1%) were radiologist, 26 (3.9%) were radiology residents, 6 (0.9%) were physicists, 3 were engineers/computer scientists (0.4%).

**Fig. 2:**
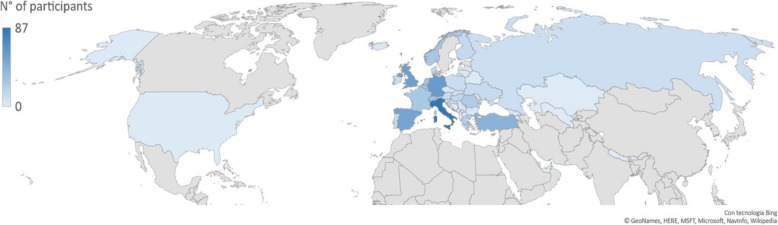
Geographic distribution of survey responders

Considering the working place, 341 responders (50.5%) work at universities/teaching hospitals, 214 (31.7%) in hospitals, 78 (11.6%) as private practitioners, 26 (3.9%) for private companies, 6 (0.9%) in private research centres. Geographic distribution of responders is depicted in Fig. 2, while Table 3 reports the number of responders per country.

**Table 3:** Survey responders by country

Country	Responders	
	Number	Percentage
Italy	87	12.9%
Germany	59	8.7%
United Kingdom	56	8.3%
Spain	52	7.7%
Turkey	41	6.1%
Netherlands	30	4.4%
Switzerland	29	4.3%
Sweden	28	4.1%
Belgium	27	4.0%
France	26	3.9%
Norway	26	3.9%
Romania	23	3.4%
Greece	19	2.8%
Denmark	15	2.2%
Hungary	15	2.2%
Portugal	15	2.2%
Finland	13	1.9%
Austria	12	1.8%
Ukraine	12	1.8%
Ireland	11	1.6%
Poland	10	1.5%
Russia	9	1.3%
Slovenia	8	1.2%
Croatia	7	1.0%
Slovakia	7	1.0%
Czech republic	5	0.7%
Georgia	4	0.6%
Latvia	4	0.6%
Serbia	4	0.6%
Bulgaria	4	0.6%
Estonia	3	0.4%
Bosnia and Herzegovina	2	0.3%
Iceland	2	0.3%
Kosovo	2	0.3%
Lithuania	2	0.3%
Macedonia	2	0.3%
Montenegro	2	0.3%
Belarus	1	0.1%
USA	1	0.1%
Total	675	100.0 %

### AI and medical imaging

**Fig. 3:**
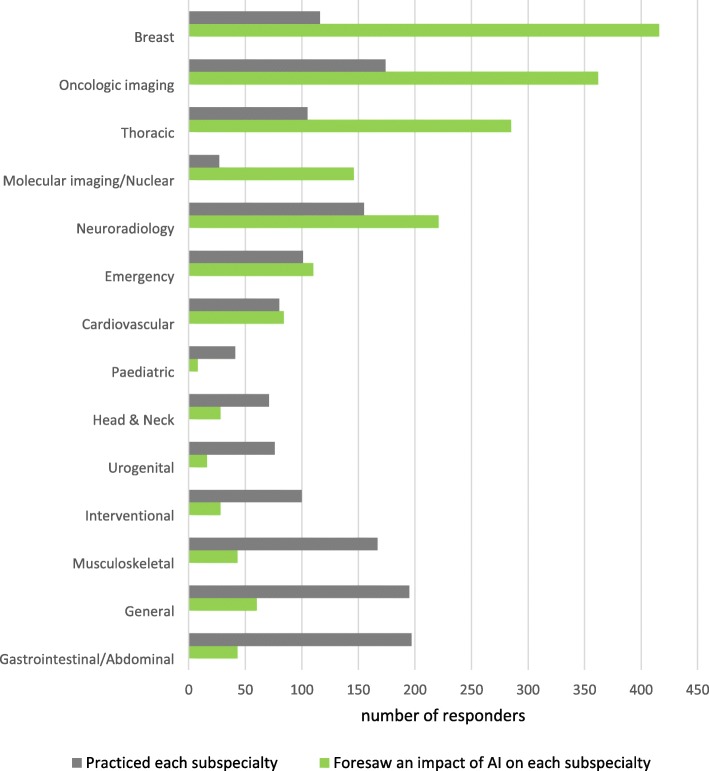
Distribution of responders. The grey bars represent the number of responders that practice each subspecialty while the green bars represent those who foresaw an impact of AI on each subspecialty. Subspecialties are sorted according to the difference between values of green and grey bars

**Fig. 4:**
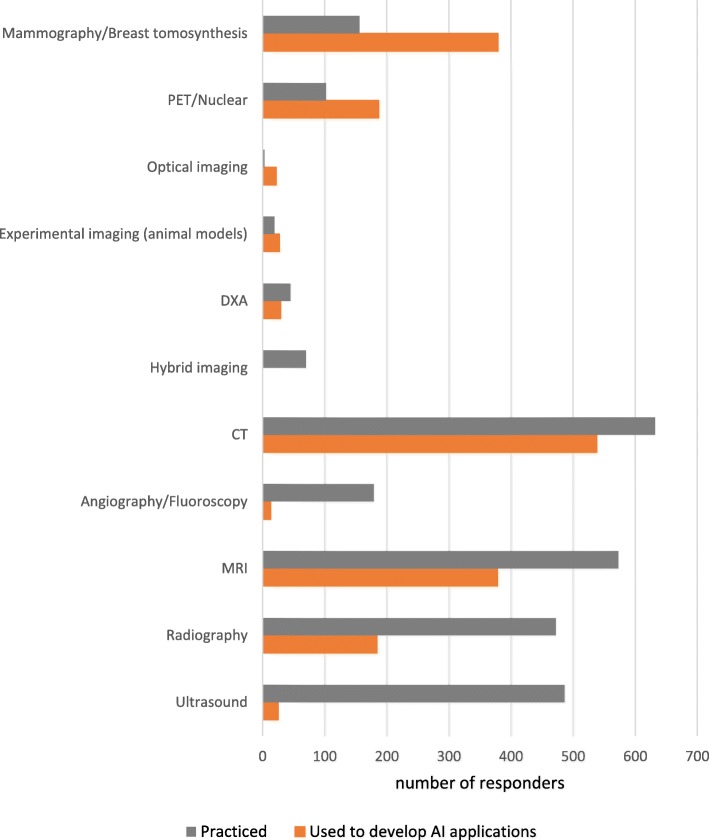
Distribution of responders. Grey bars represent the number of responders that practiced each imaging modality, while the orange bars represent those who believe that that modality will be used to develop AI applications. Imaging modalities are sorted according to the difference between values of orange and grey bars. PET: positron emission tomography; DXA: dual X-ray absorptiometry; CT: computed tomography; MRI: magnetic resonance imaging

The distribution of responders according to the practiced radiology subspecialty and their opinion about which subspecialties will be mostly influenced by the introduction of AI systems is reported in Fig. 3. In the same way, Fig. 4 shows responders’ distribution according to practiced imaging modalities and their opinion about which imaging modality will be used most to provide input data for AI systems. Table 4 shows AI applications in radiology and their corresponding rates by responders.

**Table 4:** Applications of artificial intelligence in radiology and their corresponding rates by 675 responders.

Applications	Preferences	Percentage
Detection in asymptomatic subjects (screening)	406	60.1%
Staging/restaging in oncology	314	46.5%
Quantitative measure of imaging biomarkers	256	37.9%
Image post-processing	242	35.9%
Support to structured reporting	188	27.9%
Lesion characterisation/diagnosis in symptomatic subjects	184	27.3%
Detection of incidental findings	156	23.1%
Imaging protocol optimisation	128	19.0%
Prognosis	59	8.7%

### AI impact on radiologist’s daily work

The majority of 675 responders foresee an impact of AI in terms of job opportunities (375, 55.6%), 218 of them (58.1%) expecting an increase in job opportunities, the remaining 157 (41.9%) expecting a reduction. Similarly, of 675 responders, 504 (74.7%) expect an impact in terms of total reporting workload, with 256 (50.8%) of them expecting a reduced reporting workload and 248 (49.2%) expecting the opposite scenario.

More than half of 675 responders (364, 53.9%) think that AI-based application will make the radiologist’s profile more clinical, opposed to those who expect the radiologist’s profile to become more technical (187, 27.7%). Finally, 96 (14.2%) believe that the radiologist’s profile will remain unchanged.

If asked about the ability of AI-system to help to report examinations outside the field of subspecialisation, 283 (41.9%) believe that radiologists will be more subspecialty-focused, 121 (17.9%) expect radiologists to be less subspecialty-focused on, and 271 (40.1%) declare that the rate of dedication to subspecialties will remain unchanged.

**Fig. 5:**
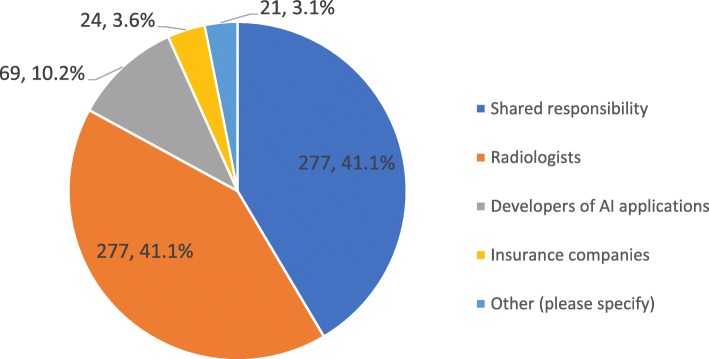
Distribution of answers related to who should take legal responsibility of AI systems outcome

Talking about who will take the legal responsibility of AI systems outcome, Fig. 5 summarises the answers, with radiologist being seen as the only responsible by 41% of responders and a scenario of shared responsibilities favoured by 41% of responders. Of the 675 responders, 374 (55.4%) believe that patients will not accept a report made by an AI-application alone without supervision and approval by a physician, while 79 (11.7%) claim the opposite; on the other hand, 222 (32.9%) believe it is now too early to estimate patients’ reaction. Among the 675 responders, 415 (61.5%) think that the use of AI systems will change the relationship between the radiologist and the patient. Among them, 275 (66.3%) believing it will become more interactive and 140 (33.7%) claiming that it will become more impersonal. The remaining 259 of 675 (38.4%) answered that the relation between the radiologist and the patient will be unchanged. Moreover, responders believe that if AI systems will allow to save time, it should be used to interact with: other clinicians (437, 64.7%), patients (322, 47.7%), AI developers (118, 17.5%), other radiologists (97, 14.4%), administrators (11, 1.6%), or other professionals (47, 7.0%).

### Radiologists’ involvement in AI systems development

Among 675 responders, 100.0% believe that radiologists will play a role in the development and validation of AI applications to medical imaging. Concerning the radiologists’ role in this process, the majority believes that they should supervise all development stages of an AI system applied to radiology (434, 64.3%). Specific tasks were rated as follows: helping in task definition (359, 53.2%), providing labelled images (197, 29.2%), developing AI-based applications (188, 27.9%). However, 390 of 675 responders (57.8%) are currently not involved in research projects on AI applications development, 158 (23.4%) are planning to be involved, 74 (11.0%) are currently involved in AI systems development, and 61 (9.0%) in their testing.

Regarding the current use of AI applications, 321 of 675 responders (47.6%) do not use them in their clinical practice, 138 (20.4%) are currently using these systems, and 205 (30.4%) do not use them at present but are planning to do it. Finally, 463 of 675 responders (68.6%) would like to be educated on advantages and limitations of AI applications, 392 (58.1%) on the clinical use of AI applications, 228 (33.8%) on how to get into the driver’s seat in using AI, 119 (17.6%) on technical methods, 75 (11.1%) on how to survive to the AI revolution, and 6 (0.9%) on how to avoid the use of AI applications.

## Discussion

In one month, 675 ESR members, most of whom are radiologists aged from 30 to 60 years, working in academic or non-academic hospitals, completed the proposed survey. The total number of responders is small if compared to the total number of ESR members. However, the number of responders per country correlates to the number of ESR members per country. The prevalence of colleagues based in academic/teaching hospitals (over 50%) should be related to the higher sensitivity to AI innovation in these centres.

Radiologists who answered the survey practiced different subspecialties. Among possible options, none prevailed. General and abdominal imaging were the most practiced subspecialties by responders, each of the two accounting for 29% of responders (see Fig. 3). In spite of the subdivision into different radiological areas, we observed a quite large agreement among them regarding which subspecialties will be more influenced by AI applications. Indeed, more than half of responders stated that breast (61.6%) and oncologic imaging (53.6%) will be most impacted by the AI revolution, followed by thoracic imaging (42.2%), neuroradiology (32.7%), and nuclear imaging (21.6%). This is reasonable, because consolidated screening programs provide, since the beginning of AI era in the healthcare, a large amount of digital data. This condition makes breast imaging the first candidate for the application of machine learning algorithms [5]. It does not surprise that the highest rated imaging subspecialties are those that frequently involve tumour detection and characterisation. Indeed, this task represents a classification problem, which is prone to be solved using machine learning algorithms [7]. Notably, radiomics represents the most promising approach for characterisation of solid cancers, which are spatially and temporally heterogeneous [8, 9]. Machine learning algorithms take advantage of the heterogeneity of imaging data used for cancer patient diagnosis and treatment. Imaging protocols traditionally used for cancer patient management include both morphological and functional imaging, which can be successfully processed using machine learning algorithms. These algorithms take advantage from the volume and heterogeneity of information contained therein to detect specific patterns starting from raw data.

Most of responders practice CT and MRI, accounting respectively for 93.6% and 84.9% of responders. Other frequently practiced modalities are US (72.0%) and radiography (69.9%). As expected, when asked to foresee which imaging modalities will be the target field of application of AI systems, responders suggested CT (79.7%) followed by MRI and mammography (56.0 % each). We should note that CT and MRI are only relatively standardised modalities while mammography (at least in the screening setting) is highly standardised. Conversely, US remains poorly standardised in every field, adding a confounding variability to the data that machine learning should model (except for automated breast US, still in its investigational phase). In fact, US was not selected as a probable field of AI application in the next 5–10 years: only 3.7% of responders foresee AI systems implementation on US practice.

It does not surprise that many responders suggested that the detection of disease in asymptomatic subjects (60.1%), staging/restaging in oncology (46.5%), quantitative imaging biomarkers (37.9%), and image postprocessing (35.9%) should be regarded as target application of AI systems. In support of this claim, it should be noted that machine learning algorithms showed valuable alternative to conventional image processing approaches in several tasks, such as image segmentation, registration, classification, and enhancement, making them the most promising approach in computer vision and medical image processing [4, 10].

Radiologists foresee an impact of AI systems on their job opportunities (55.6% of responders) and reporting workload (74.7%). However, whether this impact is felt as an increase or a decrease is unclear: responders forecast both scenarios with similar probabilities (58.1% versus 41.9% for increase/decrease in job opportunities¸ 50.8% versus 49.2% for increase/decrease in reporting workload). This fact points out radiologists’ uncertainty about their professional future in the AI scenario. At present, there is no agreement on how AI will affect the labour market or the workload of a radiology department due to the currently limited application of AI systems in daily clinical practice. In fact, only 20.4% of survey responders are currently using AI-based application in their practice. Curtis P. Langlotz [11] recently documented how the predictions of a fast disappearance of radiologists from the healthcare systems (“the mirage of job displacement”) are very far from real clinical practice for now and the predictable future for many reasons, including the “long tail” of the disease distribution, accounting for rare diseases. Taking in mind the example of the autopilot for human pilots, he repeated the simple true affirmation: *Radiologists who use AI will replace radiologists who don’t*.

The percentage of responders believing that AI will make their duties more clinical (53.9%) or unchanged (14.2%) is higher than those who foresee a more technical profile (27.7%). In addition, the percentage of responders believing that AI will make radiologists more subspecialty-focused (41.9%) or not different from now (40.1%) is higher than those who foresee a less subspecialty focus (17.9%). Thus, AI is mostly perceived as an innovation favouring a higher clinical profile and subspecialty dedication of radiologists, which are key factor for being visible to colleagues and patients, for instance playing a pivotal role in multidisciplinary tumour boards. In fact, the opposite AI-driven scenario would be radiological reporting as a low-value commodity.

As a further demonstration of a positive perception of AI by the responders, they consider other clinicians (64.7% of responders) and/or patients (47.7% of responders) the main target for using the time potentially saved by using AI, thus increasing the quantity and quality of clinical communication with them. In fact, 40.7% of responders think that AI will make the radiologist-patient relation more interactive and only 20.7% think that AI will make the radiologist-patient relation more impersonal (38.4% do not foresee any change). Overall, there is a prevalence in favour of AI as a factor making the radiologist’s role more clinical and patient-oriented.

The debate about who will take the responsibilities of AI outcome is controversial [12]. Of note, this is a central issue because it may drastically affect the role of radiology and radiologists in the healthcare systems. The results of the survey are somehow unexpected. On the one side, 55.4% of responders believe that patients will not accept a report made by an AI-application alone (without supervision and approval by a physician) and only 11.7% claim the opposite (32.9% believe it is now too early to estimate patients’ reaction). However, only 41.1% of responders identify the radiologists as the only professionals who will take the responsibility of AI outcome, 41.1% would favour a “shared responsibility”, with a minority of responders proposing AI developers (10.2%), or insurance companies (3.6%). We interpret this data as a word of cautiousness. We are at the beginning of this revolution. To take the responsibility of AI outcome implies many things up to now not clearly defined, first of all the potential for modulating the AI outcome in relation to the specific patient, to correct or declare a different opinion, to discuss with other clinicians a given outcome (think about the imaging-based declaration of disease progression during an anticancer treatment). These results clarify that accountability of AI-systems output still represents an open challenge that will require regulations at the European level and across the countries.

An important result of this survey is that 100% of responders believe that radiologists must be involved in AI-system development and validation. Radiologists are eager to be protagonists of this revolution and to manage all the steps from the development to the application of AI systems. To reach this scope, they suggest that training programs should be adopted to teach trainees and clinical radiologists advantages, limitations, and clinical use of AI-based systems. Unfortunately, only 30% of them considers the hypothesis to play a key role in the creation of labelled dataset. As a matter of fact, the accuracy of data-driven AI systems strictly depends on data quality [13]. Without radiology expertise, to expect an accurate outcome and consequently a successful translation of AI systems from research setting to clinical practice is only a wishful thinking. The radiologist’s role in this technological revolution is fundamental. Data-driven approaches require someone that ensures the quality of data, especially when supervision is needed during training of machine learning models. In addition, radiologists’ expertise in diagnosis, treatment planning and prognosis, and related issues, is the cornerstone of any successful translation of developed solution from research to clinical practice. Radiologists should flank engineers, computer scientists, and other people involved in the development of a decision support system, with emphasis on task definition and high-quality image labelling.

This report has two main limitations, already mentioned at the beginning of Discussion. First, the limited number of responders compared to the ESR members and the number of members who were reached by the emailing system. Then, half of responders work at university or teaching hospitals, so the answers reflect the opinions and feelings of advanced realities, not of the general world of radiologists. However, the analysed answers give an idea of that part of the radiological world that has a guiding role and also teach radiology to residents who are the future of radiology.

In conclusion, the responders to this survey showed on average a favourable attitude towards the adoption of AI-systems. A positive scenario of a more clinical and patient-centred radiological profile in the AI environment is foreseen. However, radiologists still do not know how fast and how disruptive the implementation of machine learning systems in radiology will be. This explains some cautious answers on the role of radiologists in responsibility for AI outcome and in the development of AI systems, considering that many ethical and legal issues related to the use of these systems are still unsolved. The future will depend on what we are doing now and what we will do in the near future, *i.e.*, on our ability to exploit the many opportunities that AI will offer to radiology and radiologists [14]. As Alan Key said: *The best way to predict the future is to invent it* [15].

## Data Availability

The datasets generated and/or analysed during the current study are not publicly available but are available from the ESR on reasonable request.

## Abbreviations

AI: Artificial intelligence
CT: Computed tomography
DXA: Dual X-ray absorptiometry
EIBIR: European Institute for Biomedical Imaging Research
ESR: European Society of Radiology
EuroAIM: European Network for the Assessment of Imaging in Medicine
MRI: Magnetic resonance imaging
PET: Positron emission tomography
US: Ultrasound
